# Short GSM mobile phone exposure does not alter human auditory brainstem response

**DOI:** 10.1186/1471-2458-7-325

**Published:** 2007-11-12

**Authors:** Gábor Stefanics, Lóránd Kellényi, Ferenc Molnár, Györgyi Kubinyi, György Thuróczy, István Hernádi

**Affiliations:** 1 Department of Experimental Zoology and Neurobiology, University of Pécs, Hungary; 2 Department of Non-ionizing Radiation, National "Frédéric Joliot-Curie" Research Institute for Radiobiology and Radiohygiene, Budapest, Hungary; 3 Institute for Psychology, Hungarian Academy of Sciences, Budapest, Hungary

## Abstract

**Background:**

There are about 1.6 billion GSM cellular phones in use throughout the world today. Numerous papers have reported various biological effects in humans exposed to electromagnetic fields emitted by mobile phones. The aim of the present study was to advance our understanding of potential adverse effects of the GSM mobile phones on the human hearing system.

**Methods:**

Auditory Brainstem Response (ABR) was recorded with three non-polarizing Ag-AgCl scalp electrodes in thirty young and healthy volunteers (age 18–26 years) with normal hearing. ABR data were collected before, and immediately after a 10 minute exposure to 900 MHz pulsed electromagnetic field (EMF) emitted by a commercial Nokia 6310 mobile phone. Fifteen subjects were exposed to genuine EMF and fifteen to sham EMF in a double blind and counterbalanced order. Possible effects of irradiation was analyzed by comparing the latency of ABR waves I, III and V before and after genuine/sham EMF exposure.

**Results:**

Paired sample t-test was conducted for statistical analysis. Results revealed no significant differences in the latency of ABR waves I, III and V before and after 10 minutes of genuine/sham EMF exposure.

**Conclusion:**

The present results suggest that, in our experimental conditions, a single 10 minute exposure of 900 MHz EMF emitted by a commercial mobile phone does not produce measurable immediate effects in the latency of auditory brainstem waves I, III and V.

## Background

There are about 1.6 billion GSM mobile phones (MPs) in use throughout the world today. Due to the close proximity of the antenna of the mobile handset to the user's ear and head, the brain is inevitably exposed to EMFs with a relatively high specific absorption ratio (SAR). Results of experimental radiofrequency (RF) dosimetry indicate that approximately 40–55% of the mobile phone's RF output power energy is absorbed in the users head [1]. Due to the high number of MP users and the relative high SAR close to the ear, it is important to resolve whether or not EMF exposure by MPs can adversely affect the human hearing system.

Numerous studies have investigated the electrophysiological effects of EMF exposure in humans. A significant delay in the latency of the fifth wave (V) of the ABR after 15 min of exposure to EMF emitted by a GSM MP was recorded [2] and the authors suggested that the observed delay might lead to a temporary 15–18 dB hearing deficiency above 2 kHz in the normal hearing frequency range. On the other hand, a different study found no effects on ABR (I, III and V waves) after a 30 minute MP irradiation [3]. Studying the influence of MP EMFs on outer hair cell functions [4] revealed, that a single 10 min MP exposure did not induce any changes in the generation of distortion product otoacoustic emissions (DPOAE) in humans. Investigation of hearing threshold levels by pure tone audiometry and transient evoked otoacoustic emissions before and immediately after 10 min of genuine or sham exposure of MP EMF yielded no significant effect caused by the irradiation [5]. These results suggest that a single 10 minute exposure to EMF emitted by a MP has no immediate effects on hearing threshold levels. Investigation of the effects of EMF on later auditory event-related brain potentials also yielded controversial results. No significant effects of 30 min of MP exposure were found on middle latency responses [3]. The authors concluded that 30 min of MP irradiation had no short-term adverse effects on human auditory system. However, in an auditory oddball task, a significant decrease in the amplitude and latency of N100 component of the auditory evoked response to non-target stimuli and an increase in the latency of the P300 to target stimuli were found as a result of one hour MP EMF exposure [6]. In this study, reaction times were significantly slower during EMF exposure, and the authors suggested that MP exposure might affect neuronal activity and alter human cognitive performance. However, a more recent study from the same group [7] investigated the possible effects of 30 min MP EMF on auditory and visual evoked responses in 120 normal subjects. In this follow-up study, previous positive findings were not replicated and the authors concluded that there was no evidence for any effects of acute MP EMF exposure on event-related brain potentials and reaction time in humans. In a recent study [8], somatosensory evoked potentials were recorded in 12 normal subjects before and after exposure to 30 minutes of MP EMF. The authors found that somatosensory cortex was not affected by exposure to EMF. Facilitating effects EMF emitted by MPs on human cognitive functions have been reported by several authors. A decrease in reaction time was observed in a choice reaction time task [9], in simple reaction time and vigilance tasks [10] and in a memory task during high memory load [11]. However, subsequent studies could not replicate these findings [12,13]. EMF exposure was reported to have significant effects on human brain oscillatory activity in the 8–10 Hz frequency band during a memory task [14], but memory performance was found similar during sham and genuine EMF exposure. Effects of MP EMF on pre-attentive processing and working memory were studied by event-related brain potentials during a working memory test [15]. The authors observed a significant effect of EMF exposure on the P50 component, whereas in their study memory performance was also not affected by EMF exposure.

Considering the possible biological effects of MPs used widely in daily communication as a high-priority environmental health issue, the European Commission (5th Framework Program for Research and Technological Development) launched the GUARD project: "Potential Adverse Effects of GSM Cellular Phones on Hearing" with the aim of addressing the potential effects of GSM MP exposure on the hearing system of laboratory animals and humans.

## Methods

### Subjects

The present experiments were carried out on 30 healthy volunteers (aged 24 ± 5 years, 15 women) with no clinical evidence of hearing disorders. The protocol of the study was approved by the Ethics Committee of the University of Pécs. All subjects gave their written informed consent after the nature of the experiment had been fully explained.

### Stimuli

We delivered three types of acoustic stimuli to the subjects: condensation, rarefaction and alternating 100 μs click sound stimuli. With this protocol we also aimed to test for possible different effects of EMF exposure on brainstem response to the above types of acoustic stimuli. We delivered 2048 clicks for each stimulus type twice to ascertain their reproducibility. The stimulus rate and intensity were set to 27 Hz and 80 dB SPL, respectively. Click stimuli were generated by a loudspeaker embedded in a 34 cm long sound tube causing an acoustic delay of 1 ms to separate the loudspeakers electromagnetic artifacts from brainstem responses. The acoustic delay tube had a sound damping textile lining on the inside surface attenuating acoustic reflection noises.

### Exposure setup and Specific Absorption Rate (SAR)

During auditory brainstem response (ABR) recording and exposure subjects lay supine in a dimly lit, sound attenuated room on an electrically shielded bed with eyes closed. They were instructed to avoid unnecessary movements. Exposure was administered by means of a standard Nokia 6310 MP via external software control at a constant 2 W peak power for 10 min. The MP was mounted on a plastic headset in normal use position. In order to evaluate and control the levels of EMF exposure, the Specific Absorption Rate (SAR, W/kg) was previously assessed for the inner ear region in a brain tissue equivalent liquid phantom device. Details of the SAR measurement procedure were described elsewhere [4]. Maximum peak SAR at a distance of 3 cm from the surface of the phantom, corresponding approximately to the position of the cochlea, was 0.41 W/kg at 900 MHz frequency.

### Audiometry

The hearing status of participants for each ear was measured for both air (125 Hz to 10 kHz) and bone (250 Hz to 2 kHz) conducted sound stimuli. A clinical audiometer (Medicor ATK-5-N20-10-84) was used to obtain standard audiograms for all subjects. A high quality headphone (Telephonics Corporation P/N OC017) was used for auditory stimulus delivery. Hearing threshold levels (HTL) in both ears of 30 recruited subjects were no worse than 30 dB at the standard audiometric frequencies.

### ABR recording conditions

Subjects were randomly assigned to one of the following groups: EMF group (fifteen subjects exposed to genuine EMF irradiation, eight women), Control group (fifteen subjects exposed to sham EMF irradiation, seven women). According to our experimental protocol, the administration of genuine or sham exposure was double blind. Our study was carried out in close agreement with the protocol of the European Commission 5th Framework project "GUARD: potential adverse effects of GSM cellular phones on hearing [16]."

### ABR recording and data analysis

The auditory brainstem response (ABR) was recorded with three non-polarizing Ag-AgCl electrodes. Electrode impedances were kept below 5 kOhms measured at 15 Hz. All electrodes were filled with standard EEG paste (TEN20, Weaver and Co., Aurora, CO). Resting DC electrode potentials were measured by an impedance meter. Differential potentials of the electrode pairs were set to less than 1 mV.

The active electrode was placed on the right mastoid and the reference electrode was placed over the vertex (Cz of the international 10–20 system). The ground electrode was placed on the forehead over the nasion and was connected to the active ground of the amplifier. The amplifier was set to a gain of 10 k. The lower and upper cut-off frequencies were set at 100 Hz and 3000 Hz, respectively.

Continuous EEG signal was recorded with a sampling rate of 20 kHz at 12 bit resolution (CED 1401, Cambridge Electronic Device Ltd, Cambridge, UK) and stored on a hard disk. Data processing and analysis was performed off-line with a custom-built MATLAB software routine written by one of the authors (GS) on a personal computer.

For each stimulus, an epoch of 15 ms duration including a 3 ms pre-stimulus period was extracted from the continuous EEG data. Epochs with a potential change below 0.1 μV or above 100 μV were rejected from further analysis. Due to excessive muscle artifacts, three data sets were excluded from further analysis.

Latencies of wave I, III and V were measured for each stimulus type and subject. We studied the effects of stimulus condition on these values obtained before and after EMF exposure with paired Student's t tests using the Statistica software package (StatSoft).

## Results

Tables 1, 2, 3 show the results of the statistical analysis and the mean latencies (± standard deviation) of ABR wave I, III and V before and after MP EMF exposure for the three stimulus condition. We found no significant effects of genuine/sham EMF exposure in any of the stimulus conditions.

**Table 1:** Mean ABR peak I latencies before and after *genuine *or *sham *RF exposure

**Condition**	**Stimulus type**	**n**	**Before RF (ms)**	**After RF (ms)**	**t**	**P**
Genuine EMF	Rarefaction	13	1.67 ± 0.14	1.66 ± 0.15	0.34	0.737
	Condensation	13	1.69 ± 0.18	1.65 ± 0.19	1.18	0.260
	Alternating	13	1.79 ± 0.09	1.78 ± 0.12	1.06	0.310
Sham EMF	Rarefaction	15	1.61 ± 0.21	1.63 ± 0.19	-0.50	0.624
	Condensation	14	1.66 ± 0.09	1.68 ± 0.14	-0.24	0.807
	Alternating	14	1.74 ± 0.10	1.76 ± 0.11	-1.23	0.239

Mean ABR peak I latencies (± standard deviation) before and after *genuine *or *sham *10 min 900 MHz RF exposure generated by a commercial MP and results of the statistical analysis.

**Table 2:** Mean ABR peak III latencies before and after *genuine *or *sham *RF exposure

**Condition**	**Stimulus type**	**n**	**Before RF (ms)**	**After RF (ms)**	**t**	**p**
Genuine EMF	Rarefaction	13	4.08 ± 0.19	4.11 ± 0.18	-0.52	0.610
	Condensation	13	4.04 ± 0.10	4.05 ± 0.15	-0.22	0.827
	Alternating	13	4.13 ± 0.12	4.15 ± 0.13	-0.75	0.468
Sham EMF	Rarefaction	15	4.03 ± 0.21	4.04 ± 0.23	-0.52	0.607
	Condensation	14	3.99 ± 0.22	4.01 ± 0.12	-0.40	0.690
	Alternating	14	4.02 ± 0.16	4.02 ± 0.17	-0.06	0.947

Mean ABR peak III latencies (± standard deviation) before and after *genuine *or *sham *10 min 900 MHz RF exposure generated by a commercial MP and results of the statistical analysis.

**Table 3:** Mean ABR peak V latencies before and after *genuine *or *sham *RF exposure

**Condition**	**Stimulus type**	**n**	**Before RF (ms)**	**After RF (ms)**	**t**	**p**
Genuine EMF	Rarefaction	13	5.89 ± 0.16	5.93 ± 0.13	-1.67	0.120
	Condensation	13	5.81 ± 0.09	5.83 ± 0.15	-0.51	0.613
	Alternating	13	5.85 ± 0.11	5.88 ± 0.14	-0.78	0.450
Sham EMF	Rarefaction	15	5.75 ± 0.17	5.76 ± 0.18	-0.48	0.634
	Condensation	14	5.76 ± 0.19	5.72 ± 0.15	0.41	0.684
	Alternating	14	5.81 ± 0.18	5.82 ± 0.15	-0.40	0.696

Mean ABR peak V latencies (± standard deviation) before and after *genuine *or *sham *10 min 900 MHz RF exposure generated by a commercial MP and results of the statistical analysis.

**Figure 1:**
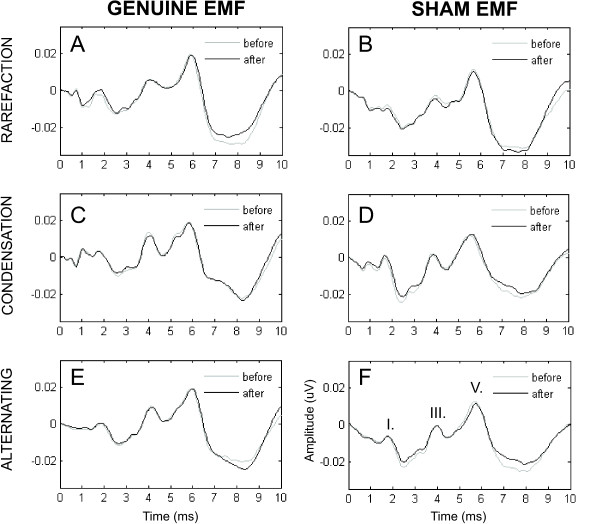
**Grand average ABR waveforms recorded before and after *genuine *or *sham *RF exposure**. Grand average ABR waveforms recorded before and after *genuine *or *sham *RF exposure for rarefaction (A), condensation (C) and alternating (E) stimuli. Panels on the left show ABR waveforms recorded before and after *genuine *EMF exposure, while panels to the right depicts ABR waveforms recorded before and after *sham *EMF exposure for rarefaction (B), condensation (D) and alternating (F) stimuli.

Figure 1 demonstrates the grand average ABR waveforms recorded in the genuine and sham EMF exposure conditions by rarefaction, condensation and alternating stimuli.

## Discussion

In this study, we found no significant effects of 10 min genuine MP EMF exposure on the latencies of wave I, III and V of the auditory brainstem response for rarefaction, condensation or alternating stimuli. Our current results are indirectly confirm the results of earlier investigations demonstrating that 10 minutes of GSM MP exposure does not induce measurable changes in cochlear function [5] in humans, possibly resulting in no deficiencies in the functioning of the central auditory pathways.

The present results reporting no adverse effects are also in line with previous negative results obtained by a similar technique [3]. The authors there studied ABR, and the ABR recovery function as well as middle latency response before and after using a MP for 30 minutes at 0.8 W power in 15 normal hearing volunteers and found none of these measures affected by exposure to EMF. In a different study [17], the authors investigated the effects of 20 minutes EMF generated by MP on the ABR before, during and after the exposure in 45 young, healthy volunteers. Consistently with our current results, they observed no changes in the latency of waves I, III and V during and after exposure to EMF compared to the initial ABR response.

Contrary, these results are inconsistent with those of a previous pilot study from our laboratory [2], where a single 15 minute MP EMF exposure at maximal 2 W output power was found to cause a significant delay of 0.207 ms in the latency of wave V of the ABR evoked by 80 dB alternating polarity clicks at 27 Hz stimulus rate. The authors there suggested that the observed delay might have been caused by the altered functioning of the exposed cochlea due to the absorbed RF energy. However, in the present study, we failed to replicate these previous positive findings. However, in the current study, we administered 10 min of irradiation at 2 W output power, whereas in the previous study [2] the irradiation lasted for 15 minutes at 2 W output power. This means that the energy absorbed in the head of the subjects in the present study was about 2/3 of that in the previous study. The shorter irradiation may also account for the present negative findings. In addition, in the previous study, the total number of participants was 10 while in the current study 15 subjects were included in each group, making the statistical power of the current data higher. Taken together, as findings of other studies [3,17] also showed no adverse effects of EMF exposure, we conclusively claim that a single 10 minute GSM MP exposure does not induce significant changes in human ABR. Nevertheless, due to the different exposure conditions of the studies which yielded negative results [[3,17]], the previous positive results [2] must still be taken with caution and indicate the need for additional control experiments. In future studies concerning cochlear microphony it would also be important to investigate any possible effects of EMF exposure on the functions of the cochlea.

## Conclusion

Our present results demonstrated that a single 10 minute-exposure to EMF from commercial GSM MPs does not induce measurable effects in ABR peak latencies and may not cause hearing loss, as it was also proposed elsewhere. However, as the potential adverse effects of longer or chronic EMF exposures have not yet been systematically tested, additional experiments are needed to reveal any possible adverse effects of EMFs on the hearing system of humans. In addition, as new generations of mobile sources of EMFs (e.g., 3 G system phones) are being rapidly introduced, it is of utmost importance to establish whether or not these new generations of MPs have potential adverse effects on brain functions in humans.

## Competing interests

The author(s) declare that they have no competing interests.

## Authors' contributions

LK and GT participated in study design. GS and LK have collected the data. FM, GK and GT performed SAR measurements. GS and IH analyzed the data. GS, IH and GT wrote the paper. All authors have read and approved the final manuscript.

## Pre-publication history

The pre-publication history for this paper can be accessed here:


